# Disease perception and experiences among rural Bangladeshi hypertensive women: A qualitative approach

**DOI:** 10.15171/hpp.2020.11

**Published:** 2020-01-28

**Authors:** Yasmin Jahan, Michiko Moriyama, Md Moshiur Rahman, Kana Kazawa, Mariko Mizukawa, Atiqur Rahman, Abu Sadat Mohammad Sayeem Bin Shahid, Sumon Kumar Das, Abu Syed Golam Faruque, Mohammod Jobayer Chisti

**Affiliations:** ^1^Graduate School of Biomedical and Health Sciences, Hiroshima University, Hiroshima, Japan; ^2^Division Ageing and Social Change (ASC), ISV, Linköping University, Linköping, Sweden; ^3^International Centre for Diarrhoeal Disease Research, Dhaka, Bangladesh; ^4^Child Health Division, Menzies School of health Research, Northern Territory, Australia

**Keywords:** Perception, Experience, Understanding knowledge, Barriers, Hypertension

## Abstract

**Background:** Hypertension (HTN) is well established as a leading cause of common serious illnesses worldwide. We carried out this qualitative research to understand perception of and experiences related to HTN among rural Bangladeshi hypertensive women.

**Methods:** A total of 74 female hypertensive participants who were diagnosed as HTN were purposively recruited in a rural community in Mirzapur, Bangladesh. A focus group discussion(FGD) was applied to share their perception and experiences. Transcripts were read in an iterative process, and a thematic analysis was performed. This paper is reported followed by COREQ checklist.

**Results:** Three main themes were generated; (i) Perception of HTN based on experiences, (ii)Knowledge of management of HTN, and (iii) Barriers of management of HTN. Under the themes, seven subthemes were identified. The participants only knew about their high blood pressure(HBP) when they had symptoms, and they applied traditional remedies in the rural context to deal with those symptoms. Even though more than half of participants had relevant knowledge of how to manage HTN, but still there were social-cultural and economic barriers and lack of social infrastructure to access healthcare, existed to practice them.

**Conclusion:** Based on our study reports, health education programs at the household and community level could be a potential starting point for any preventive and containment strategy in rural communities of Bangladesh.

## Introduction

Hypertension (HTN) remains one of the foremost non-communicable diseases (NCDs),^1^ that most often leads individuals towards cardiovascular diseases (CVDs) and its diverse complications such as stroke, kidney failure, disability even premature death.^2-4^ It has already been recognized as a leading global public health threat, ranking third as a cause of disability-adjusted life-years (DALYs).^5^

According to the World Health Organization (WHO), nearly 17 million deaths that occur worldwide due to CVDs, HTN alone contributes to 9.4 million deaths.^6,7^ The overall global prevalence of HTN in 2010 was 31% of the world’s adult population; 29% in high income countries and 32% in low- and middle-income countries. In particular, an estimated 1.39 billion people had HTN in 2010^8-10^ in Southeast Asia region. Like other developing countries of South Asia, Bangladesh has been observing an epidemiologic transition from communicable diseases to NCDs.^11^ For example, according to a pooled estimate about 21% of the individuals (>18 years) in Bangladesh are currently suffering from HTN.^12^

Recent studies revealed that those observations are due to continuing shortfalls in the awareness, knowledge, attitude, and perception to control HTN.^13^ In many countries, the health status especially of rural populations is generally poorer than that of urban living populations.^1^ In the rural communities of developing countries, people have also less accessibility to media and the resources of informative materials which may support poorer health care seeking behavior. The situation may be more problematic for rural people living in remote and hard-to-reach settings. Evidence shows lower levels of health indicators in rural areas, compared to their counterparts living in urban areas, more often in developing countries.^2,14^ Moreover, in recent years, Bangladesh is coming across a high risk of HTN, such disparities may emerge from poor socio-economic and environmental conditions, poor access to healthy nutrition, high exposure to health risk factors, and poor access to health care services in rural areas.^3^

HTN clinically stands for high blood pressure (BP), defined as a medical condition in which the arterial BP is elevated exceeding 130 over 80 mm Hg. This elevation challenges the functions of heart to circulate blood through the blood vessels.^15,16^ Because of the silent nature of HTN progression, it takes a long time to be diagnosed. Early diagnosis through regular check-ups and accessibility to appropriate treatment can be the key components to reduce HTN burden.^17^

Despite a substantial evidence that people in Africa and Asia die from preventable diseases,^5^ health education has been suggested as a preventive tool for majority of these diseases.^13^ Health education is one of the main components of healthcare services in addressing the major health concerns, like maternal and infant mortality, infectious diseases and even in healthy life promotion programmes.^6,18^ In order to provide them with health information and increase health literacy, there is a need for implementing health education programs offered by qualified heath care providers.^4^

The purpose of this study was to reveal individuals’ perceptions of, and experiences related to HTN in a rural community of Bangladesh. The results can be used for development of educational materials which aim to produce perceptional and behavioral changes in the target population of our intervention study focused on HTN.

## Materials and Methods

### Study design

A focus group discussion (FGD) was conducted in a rural community of Mirzapur under Tangail district, Bangladesh during mid-June to mid-July 2018. FGD is the best way to reveal the local community perception and experiences exist in the area rather than taking one-to-one interview. It is effective for generating rich data through interaction among participants with reducing hesitation,^19^ especially low educated women in rural community context.

### Study site

Mirzapur is located about 60 km north to Dhaka, the capital city of Bangladesh. It consists of 219 villages and the total population is around 407 781 in 2011. Mirzapur has an average literacy rate of 56% (7 years and more of schooling) where male and female literacy rates are 47 % and 39% respectively. The residents are living with poverty and low school attainment.

### Study participants and sampling

Participants for the study included hypertensive individuals who were diagnosed by different health facilities like Kumudini Women’s Medical College and Hospital, private clinics and others health facilities of the locality or consulting physicians to whom they sought care. The eligibility criteria were as follows: (*i* ) women aged 35 years and more because prevalence of HTN increases from this age group,^12^ (*ii* ) diagnosed (recently or in the past) cases of HTN confirmed through review of hospital records or prescriptions, and (*iii* ) able to travel with minimal assistance to attend along with others in a group to the FGD session.

Eighty participants were invited to participate in the study by household visits with the help of community health workers (CHWs). Purposive sampling is a non-probability sampling method which enable to grasp variation range of HTN community people in local context with maximum variation in terms of age, gender, marital status, level of education and occupation. However, male individuals were reluctant to participate in the FGD sessions because of their job commitments, therefore, the present qualitative study included only women individuals in the FGD sessions.

A three-independent team-member of a clinician (1) and research assistants (2) prepared a list of 80 participants with the help of CHWs. Among them, 6 participants refused to participate. The individuals expressed their interest in participating in FGD sessions voluntarily and finally provided their informed consent in writing.

### Data collection

The first author, a female primary care physician who has practiced in the local community for five years, a doctoral student of health science major, and the sixth author, a male anthropologist (has qualitative research expertise), conducted the focus group as facilitators. The trained CHWs actively assisted in organizing the FGD sessions.

At the beginning, the primary researcher explained the issues related to HTN in the area and the purpose of doing the research, but the participants had no prior knowledge about the members of the research team. However, they aware about the research before conducting the sessions. The researchers have sound knowledge about the community level research. As they were not familiar with that community people, there were no chances of biasness, assumptions or any kinds of selected research topics.

A guideline was developed by following the research protocol based on previous literature to facilitate FGD sessions.^20^ Some of the grounding questions were:

*“What do you know and think about hypertension?”, “Can you share any of your personal experience with us?”,*

Additionally, during the discussion session some probing questions were also raised such as*what are the possible opportunities you have followed in this regard?”, “Do you think salt consumption and hypertension are associated? If yes, how did you know about it?” “Do you receive any consultation from doctors who provide treatment for hypertension?”, “Could you please tell me about your daily habit?”* to make the discussion more interactive. Since many participants stated their difficulties in practicing doctor’s recommendations and make a regular hospital/clinic visit, we asked them the reasons too.

The aim and procedure of the study were explained to the participants, and they provided written informed consent. In addition, participants were informed about the right to withdraw at any time during the interview. Participants were simply asked to share their perception of, understandings, and experiences related to HTN.

The guideline and its necessary modifications were discussed thoroughly among the research team members during extensive field-testing in rural environments (among a group of hypertensive individuals who obtained care after hospitalization, but they were not included in the present qualitative study).

Total eleven FGD sessions had been conducted, and 6–8 individuals (pre-enlisted individuals)^21^ were invited to participate in each session. The FGD sessions had been conducted in community settings at participants home. At the beginning, we gave identification number (based on random selection) to the participating individuals and wrote their name in a piece of paper. They were requested to utter their number each time before expressing their opinions. One session was arranged per one group, and each session lasted for 35–45 minutes. All the sessions were audio recorded with their permission and extensive field notes- an accurate description of what is observed- were taken concurrently based on the discussions.^22^ The time and place of interview sessions were arranged based on the locales convenient to the participants. Data collection ceased when the point of saturation was achieved, and when the researcher comprehends that the participants’ perception, understandings, and issues related to HTN. Moreover, the narrative data were found to be rich and had insightful meaning and considered that the additional data would not generate any new information.

### Data analysis 

Dara were analyzed following thematic analysis guidelines recommended by Braun et al.^23^ All discussion sessions recorded on tape were transcribed accordingly. Codes were developed form the textual data based on the differences and similarities after detailed examination of the transcribed texts. Then themes and subthemes were identified.

MS Excel software was used to manage the textual data.

### Data trustworthiness 

Data trustworthiness was ensured by following the criteria recommended by Graneheim and Lundman.^24^ The first author, doctoral student checked the interviewed data and analysis, and the sixth author, anthropologist assist the first author to check the interviewed data as well as the findings at each step of the study process. Next, the second author and fifth author both of them are expert in chronic health care, further analyzed the data and discussed the themes and subthemes with the first author to ensure auditability and dependability of the data. Along with the first author, CHWs who lives in the community, again reviewed the results to check the consistency with the local context they have experienced.

## Results

The mean age of the 74 participants was 52.2 years (35–85 years). All participants were married and housewives by occupation. More detailed information about the participants sociodemographic are presented in Table 1.

From the data analysis, three themes were identified, namely (i) Perception of HTN based on experiences, (ii) Knowledge of management of HTN, and (iii) Barriers of management of HTN. Under the themes, seven subthemes were identified. In the following, these three themes and seven subthemes are presented and discussed.

### Perception of hypertension based on experiences

*Understand high blood pressure by symptoms and being afraid of it as a cause of stroke*

All participants used a word of HTN as “*blood pressure is high”* and described its symptoms as listed in Table 2. All of them only perceived that they were in high blood pressure (HBP) condition when they had symptoms. Most of the participants mentioned headache (*something heavy get down on my head* ), neck pain (*scraping pain on my neck* ), vertigo (*everything rolling around me* ), and vomiting (*feeling heavily discomfort* ) as the symptoms of HBP. Individual’s understanding of HBP indicated that it was a serious disease for both elderly and adult population and as a cause of stroke which led to death based on their experiences.

A participant stated that: *“HBP is a bad thing. HBP causes something heavy get down on my head and scraping pain on my neck. Usually, things are rolling around me while I am having a high rise in BP, as this problem of dizziness is known to my family members, someone in the family pours water on my head at that time. I take medicine regularly, one time in a day to keep this problem under control”* .

Another participant further added that: *“HBP is a serious disease; it can cause stroke, even paralysis which leads to death”* .

Because they do not know the clinical criteria of HTN, and they did not have practice on measuring BP at home, they understood their HBP only by symptoms, and they were unconditionally afraid of serious symptoms which led severe consequence. Therefore, they often relied on local recommended coping strategies of HBP.

*First knowing that having HTN from a neighboring person, not from a doctor*

All the study participants deemed that they came to know about HBP from their family members, friends, relatives and neighbor prior to be diagnosed by a doctor. However, less than half of the participants had prior understanding of symptoms of HBP. They acquired knowledge about HTN from a sick individual of the family, friends, relatives or neighbor. Larger portion of the study participants who were not aware of symptoms of HBP became responsive once they individually started suffering from it. After neighboring person pointed the possibility of having HTN, they went to pharmacy or nearby health care facility due to their symptoms. Another participant talked her story:

*“I became aware of HBP when my mother had died of HBP. One day suddenly mother felt severe chest pain; we could not recognize what happened. That was due to HBP. My brother thought it could be due to hyperacidity and gave medicine for that problem; afterward, my mother was taken to the nearest hospital, but she died right after she had arrived at the hospital. The attending doctor said that she had a stroke that might have occurred due to HBP which remained undiagnosed and untreated for a long time”* .

### Knowledge of management of hypertension

*Limited knowledge and misunderstanding of managing hypertension*

Researcher’s further asked about the causes, mechanism, and clinical criteria of HTN, participants responded by *“increase of blood flow in the human body”.* Very few participants said that it had two values (upper and lower); if there was any rise in upper level of pressure then the condition was called HBP.

A very few participants had identified chest pain, blurred vision, sleeping disturbance, irregular heartbeat/palpitation as symptoms of HBP. Moreover, most of the participants have indicated stroke as a common complication of hypertensive disorders as well as heart attack (they explained as chest pain), paralysis and death as possible consequences of HBP. However, none of the participants had experiences about other complications of HBP such as kidney disease, eye problems and so on.

However, majority of the participants identified the major challenges regarding medication adherence regularly was ‘*their unawareness’* . The participants who perceived HBP as a temporary rapid rise of BP, take medication when they have symptoms and did not take medication when there have no symptoms. They failed to perceive the severity of illness sometimes which requires adherence to drug as well as regular follow up visits.

One participant mentioned that:

*I take medicine only when I feel bad. Moreover, I stop taking drugs when I feel good.*

*(Mis)perception regarding development and treatment of hypertension*

A common knowledge among the participants was some modifiable factors (causes) are responsible for developing HBP. According to their discussion, most of the participants had dietary knowledge related to prevention of HBP. Moreover, they had a clear idea about what food to eat and what to avoid. For example, eating green leafy vegetables daily is a worth choice in controlling HBP. More than half of the participants mentioned that eating more beef, chicken, fatty and oily foods, consumption of an excess amount of salt could cause HBP. Here are some statements of the participants:

One of the participants clearly shared that, *“I came to know about HBP from my nephew, who told me not to drink milk, and eat egg or red meat. That’s why I only eat a very little piece of red meat as HBP is a very dangerous disease and can even cause stroke”* .

Another participant stated that, *“By controlling some habits one can decrease HBP risks and doctors suggested me not to eat beef, milk, egg yolk, fatty food and extra salt”* .

Another participant deemed that, *“For being overweight, doctors advised me to avoid eating oily and fatty food, beef and mutton, not to take more salt, thus, not to increase the probability of any further elevated blood pressure”.*

However, more than half of the participants were fond of taking smokeless tobacco daily but very few of them were aware of the adverse effects of it. No one knew about the role of smokeless tobacco as a risk factor for developing HBP. Some of the participants stated that lean persons as well as fatty and overweight individuals can suffer from HBP. Very few participants mentioned that stress/tension can cause HBP and high level of cholesterol in blood may cause HBP. Moreover, some of the participants came to a common ground that individuals with physical inactivity and diabetes are more likely to develop HBP. In terms of physical activity, most of the participants undertook physical activity beyond household chores. Our study results showed that most of the participants had general perception on lifestyle, but very few of them were practicing those in their daily life. Here are some participant statements given below that originated during our facilitation:

A participant expressed that, *“…doctors suggested me not to take oily and fatty food, not to get involved in too much stress conditions and recommended to maintain a good sleeping order with going to bed on time and sleeping for a longer period without any interruption. Furthermore, I was suggested to walk for a time regularly”.*

Another participant stated that, *“…doctor suggested me not to taking oily and fatty food and walk regularly at least for 30 minutes”.*

Less than half of the participants conceded that individuals with family history of hypertensive disorder are more vulnerable to develop HBP and any one can develop HBP even at any age of her/his middle adulthood.

Despite the perception about HTN, the treatment strategy varied widely among the participants with some misconceptions. Some participants pursued allopathic treatment whereas others were dependent on local traditional remedies (i.e. black cumin, garlic, lemon, tamarind, and starfruit). Another group of participants believed that these herbal remedies can also reduce HBP related complications. According to the verbal statements of the study participants regarding the concepts of traditional remedies:

One participant stated that, *“…when I feel that my BP has elevated further, I eat lemon, tamarind, as well as put blended star fruit on the top of my head as early as possible, then I feel comfort”.*

Many participants followed the statement, adding “*pouring water on top of my head* .”

Another participant mentioned that, *“…one drug seller of a local drug store suggested me to take black cumin with betel leaf which will control my HBP”.*

*Barriers of management of hypertension*

More than half of participants stated that they did not visit healthcare center regularly. Therefore, the researcher probed to tell them about the reasons.

*Socio-cultural barrier exists for women in a local community to access to healthcare*

Furthermore, participants stated that they could not go alone to healthcare centers due to socio-cultural barriers and they needed someone preferably a male person to accompany them. Non-compliance to drugs was observed among very few participants, for that they implicated absence of husband’s cooperation or forgetfulness in timely purchasing of medicines from drug stores.

Participants stated that, *“We could not go alone to healthcare centers as our household members would not allow us to go outside alone, so we needed someone preferably a male person to attend us”.*

*“Most of the times our husbands are forgetful in timely purchasing medicine from drug stores. So that we cannot continue medication regularly.”*

*Economic barrier to access to healthcare*

The explanations for reported barriers in the local context where they were in financial hardship to travel in healthcare centers. Furthermore, females not involved in any income generation or in the poorest/poorer wealth quintiles were less likely than their male peers. So, they do not allow to expense money for their health care visits and medication adherence.

Few participants mentioned financial crisis that caused their inability to buy medicines.

Among them few participants deemed that, *“Our households’ financial condition is not well-off and medication expense is also very high. So, it is difficult to maintain my health expenses along with the household expenditure”.*

*Lack of social infrastructure to access to healthcare*

Few participants stated that they do not want to go to the health care center as it takes long time to travel and lengthy waiting time. Because they have to do a lot of work at their household. One participant mentioned that,

*“It always takes longer time to go to the health care center due to unavailable transportation. Moreover, we need to stay in a long queue because of number of patients. I have a lot of things to do at home. So, I cannot waste my time there”.*

## Discussion

The present study has indicated that majority of the participating hypertensive women individuals were more than 50 years old, about half of them were illiterate and most of them were observed to be consuming smokeless tobacco. Previous studies reported that increases of prevalence of HTN in Bangladesh.^25^ However, this study also revealed individual’s perception and experiences related to HTN and barriers that hinder them to access to healthcare in a rural community of Bangladesh. Our study found that participants only knew having HBP by symptoms and noticed the possibility from a neighboring person, but not from a doctor. The probability of stroke in coming days was their greatest fear. They suffer from lack of fact (BP data) and information about the consequences of HTN, which is necessary for closing the gap between perceptions and daily practice. of dietary practice. They were known by doctors.

However, a very few participants stated about upper and lower values of HBP level measurements. As the BP check behavior at home is not established in Bangladesh particularly in rural areas, so they do not have any idea, or any education related to this. People can only rely on symptoms to understand their BP level. It is dangerous to onset any kind of complications when symptoms arouse, and BP level is very high.

Nonetheless, our study results revealed that most of the participants had fair perception about symptoms of HTN but in case of complications, more than eighty percent knew about stroke and nearly twenty percent hypertensive individuals had familiarity about paralysis, heart attack and death. Very few respondents were aware that if hypertensive disorder is not managed properly that may cause development of complications of HTN. No other health related complications of HTN like kidney disease and eye problems were known to them. This may be attributed that many participants were not aware about HTN in their initial stage as it is usually asymptomatic. Moreover, HTN often takes a long time before it is diagnosed which may cause development of complications in the meantime as such damage of organs due to complications of HTN were not known to participants. One study mentioned that most participants had the perception that headache, dizziness and tiredness were the symptoms of HTN. However, very few participants knew that a common symptom of HTN was feeling of tightness in the chest.^26^

Moreover, general perceptions on lifestyle changes, the study reported that more than half of the participants knew that lack of appropriate dietary habit, obesity, and sedentary lifestyle and so on are the modifiable risk factors in controlling HTN, but they did not have any idea regarding smokeless tobacco consumption and its association with HTN. Additionally, they also had the insight that altering those behaviors will help in preventing complications. Most of the participants were aware that they should restrict their salt intake to control HTN but very few were observed to cut down salt in food once they were diagnosed to be hypertensive. Our study results showed that most of the participants had general perception on lifestyle, but very few of them were practicing those in their daily life; they were unaware of the significance of lifestyle modification in controlling and preventing HTN induced complications.

Most participants considered HBP as an important disease, though some of them did not take drugs regularly, they take only when they are symptomatic. Moreover, they described several informal ways that can mitigate symptoms of hypertensive emergency, which simply reflected presence of their inadequate perceptions of HTN. Participants were diagnosed as hypertensive when they were hospitalized with acute condition, visited clinicians with other health problems, and a few when their BP was measured occasionally by small drug selling store people located in marketplaces. The role of pharmacies in diagnosis and prescribing medicines for HTN in low- and middle-income settings has been well documented.^27,28^

Some of the participants have limited knowledge and misconceptions regarding treatment of HTN, they thought that herbal remedies might have some effective role in the treatment of HTN, although the researchers did not try to measure the role of these remedies. These apprehensions may be due to poor level of experience and perception among hypertensive individuals.^29^ Some studies have reported role of garlic and its use by sick individuals for a variety of cardiovascular conditions, especially hyperlipidemia. It has also been reported to have hypotensive actions. Researchers have indicated role of garlic in increasing nitric oxide production, resulting in smooth muscle relaxation and vasodilatation.^30,31^ Studies have reported an association between excess salt intake and HTN.^25^ Our study found the same result. Other studies have indicated that more than half of the participants were aware that mental stress, high cholesterol content in bloodstream and obesity were the risk factors for HTN.^32^ In addition, another study observed that although the participants were aware that stress, excess salt intake and obesity are risk factors for HTN, but they were poorly aware about the risk factors like sedentary lifestyle and smokeless tobacco intake.^33^ In our study, results corroborated with that of others.

Along with these some of the participants reported that they would continue taking medications to have symptoms or control symptoms of HTN, but once they felt better, they discontinued. However, some studies reported that there were some social or household barriers that stopped participants from visiting healthcare centers or taking medicines regularly. Sometimes they faced household financial shortage to purchase drugs, forgot to attend scheduled checkups or lack of sufficient time due to their household work, interruption in mobility due to absence of transports or male companion, and social distance between client and provider.^34-36^ As the females are not involved in any income generation or in the poorest/poorer wealth quintiles, so they have to ask for money to their household heads to purchase the medication or transportation cost to go for health checkup. Our study also has the similar findings.

However, Bangladesh has achieved dramatic success, especially over the last 20 years, in closing gender inequalities in female mortality and malnutrition. A number of community-based interventions from the government and non-government organizations including outreach sites and satellite clinics, CHWs, female education programs and women empowerment through micro-credit schemes could be attributed for such achievements.^37^ During 1993–2011, coverage of antenatal care at public health facilities, which includes routine BP measurement, doubled in Bangladesh.^38^ However, despite an increase in social standing and the increased accessibility of health system, Bangladeshi women still lack empowerment and financial freedom and gender inequity is prevalent.^39^

We have found many barriers produced from socio-cultural, poor economy, and lack of social infrastructure exist in the local community. From the above discussion, further steps can be explored the situations and discussed about strategies to overcome these situations. Especially in those countries where women’s behavior in is much influenced by men including household level. To be successful for controlling HTN, we need to work for total community including that male dominant areas.

### Strengths and limitations

Strengths of this study are its exploration of an individual’s perspective on their personal, social and cultural contextual backgrounds that would hinder them on proper management of HTN. The FGD sessions allowed us to reveal perceptions and experiences related to HTN that exists in the local community through group interaction, sharing, and integration. This qualitative study is one of the first, to our knowledge, to explore participant’s experience and perceptions about HTN in Bangladesh and attempts to capture their understandings, fear, concern, coping, and barriers. Despite its strength, this study has several limitations. All the study participants were female and housewives. They certainly represent the situation of women in this area. However, successful management of HTN at the community level requires a look at the important influencing factors of men, especially of male dominance.

## Conclusion

The study likewise distinguished numerous reasons to address the health issues like why BP control remains suboptimal, including individuals desire to maintain a sense of regularity, participant’s disinclination to monitor their own BP at home and lack of understanding of exactly what lifestyle changes to make and how to implement them. Based on that, health education programs at the household and community level or mass education campaigns could be a very good starting point to formulate appropriate preventive and control measures against HTN in rural communities of Bangladesh.

## Ethical approval

The study was approved by Bangladesh Medical Research Council (BMRC). The consent form was written in a simple local language (Bengali) so that the participants could understand the study objectives and purpose of the study easily. The consent form was read aloud to the participants if she was unable to read prior to obtaining informed written consent. Names and identities of the study participants were not used while analyzing data.

## Competing interests

The author(s) declared no potential conflicts of interest with respect to the research, authorship, and/or publication of this article.

## Funding

This study is carried out with funding of the Grants-in-Aid for Scientific Research Program (KAKENHI), Japan (No. 18H03113).

## Authors’ contributions

YJ conceptualized the study, YJ and AR contributed to the data preparation and analysis the results, drafted the original manuscript and revised the manuscript. MiM and MaM provided advice, recheck on data preparation and interpretation, MMR, KK, AR, ASMSBS, SKD, ASGF, and critically revised the manuscript. All authors read and approved the final manuscript as submitted.

## Acknowledgments

The authors would like to thank all participants who shared their perceptions and experience with us. We would also like to thank the data collectors for their efforts.

**Table 1:** Socio-demographic characteristics of the participants (N = 74)

**Variable**	**Frequency**	**No. (%)**
Age Mean=52.2	35-39	32 (43.2)
	40-44	3 (4.0)
	45-49	4 (5.4)
	50-54	6 (8.1)
	55-59	5 (6.8)
	≥ 60	24 (32.5)
Education	No education	35 (47.2)
	Incomplete primary education	4 (5.4)
	Completed primary education	8 (11)
	Incomplete secondary schooling	15 (20.2)
	Completed secondary or higher	12 (16.2)
Marital status	Married	74 (100)
Occupation	Housewife	74 (100)

**Table 2:** Local terminology for symptoms of hypertension or high blood pressure

**Terminology**	**Local Term**
Headache	*Something heavy get down on my head*
Neck ache	*Scraping pain on my neck*
Chest pain	*Something holds the chest tightly*
Vertigo/Dizziness	*Everything rolling around me*
Vomiting	*Feeling heavily discomfort*

## References

[1] World Health Organization (WHO). Global Health Observatory (GHO) data -- Raised Blood Pressure: Situation and Trends. Geneva: WHO; 2010.

[2] World Health Organization (WHO). Global Status Report on NCDs. Geneva: WHO; 2010.

[3] He J, Whelton PK (1997). Epidemiology and prevention of hypertension. Med Clin North Am.

[4] Bromfield S, Muntner P (2013). High blood pressure: the leading global burden of disease risk factor and the need for worldwide prevention programs. Curr Hypertens Rep.

[5] Lim SS, Vos T, Flaxman AD, Danaei G, Shibuya K, Adair-Rohani H (2012). A comparative risk assessment of burden of disease and injury attributable to 67 risk factors and risk factor clusters in 21 regions, 1990-2010: a systematic analysis for the Global Burden of Disease Study 2010. Lancet.

[6] World Health Organization (WHO). A Global Brief on Hypertension: Silent Killer, Global Public Health Crisis: World Health Day 2013. Geneva: WHO; 2013. p. 1-39.

[7] Lozano R, Naghavi M, Foreman K, Lim S, Shibuya K, Aboyans V (2012). Global and regional mortality from 235 causes of death for 20 age groups in 1990 and 2010: a systematic analysis for the Global Burden of Disease Study 2010. Lancet.

[8] Minh HV, Byass P, Chuc NT, Wall S (2006). Gender differences in prevalence and socioeconomic determinants of hypertension: findings from the WHO STEPs survey in a rural community of Vietnam. J Hum Hypertens.

[9] Hoang VM, Byass P, Dao LH, Nguyen TK, Wall S (2007). Risk factors for chronic disease among rural Vietnamese adults and the association of these factors with sociodemographic variables: findings from the WHO STEPS survey in rural Vietnam, 2005. Prev Chronic Dis.

[10] Neupane D, McLachlan CS, Sharma R, Gyawali B, Khanal V, Mishra SR (2014). Prevalence of hypertension in member countries of South Asian Association for Regional Cooperation (SAARC): systematic review and meta-analysis. Medicine (Baltimore).

[11] Joshi P, Islam S, Pais P, Reddy S, Dorairaj P, Kazmi K (2007). Risk factors for early myocardial infarction in South Asians compared with individuals in other countries. JAMA.

[12] Chowdhury S, Chowdhury P (2015). Prevalence of Hypertension among the Bangladeshi Adult Population: A meta-analysis of Studies between 2004 and 2014. Cardiovasc J.

[13] Spencer J, Phillips E, Ogedegbe G (2005). Knowledge, attitudes, beliefs, and blood pressure control in a community-based sample in Ghana. Ethn Dis.

[14] Hamidzadeh Y, Hashemiparast M, Hassankhani H, Allahverdipour H (2019). Local-level challenges to implementing health education programs in rural settings: a qualitative study. Family Med Prim Care Rew.

[15] Leconte M, Ismael V. Teaching Plan for High Blood Pressure Management. New York: College of Technology; 2012. p. 126.

[16] Allender JA, Spradley BW. Community Health Nursing: Promoting and Protecting the Public’s Health. Philadelphia, PA: Lippincott Williams & Wilkins; 2005.

[17] Task Force for the management of arterial hypertension of the European Society of Hypertension; Task Force for the management of arterial hypertension of the European Society of Cardiology. 2013 ESH/ESC Guidelines for the management of arterial hypertension:. Blood Press. 2013;22(4):193-278. doi: 10.3109/08037051.2013.812549. 23777479

[18] Hamidzadeh Y, Hashemiparast M, Hassankhani H, Allahverdipour H (2019). Obstacles for Iranian rural population to participate in health education programmes: a qualitative study. Fam Med Community Health.

[19] Holloway I, Galvin K. Qualitative Research in Nursing and Healthcare. 4th ed. West Sussex, UK: John Wiley & Sons, Ltd; 2017. p. 125.

[20] Guidelines for conducting a Focus group. American Journal For Researchers. Eliot & Associates; 2005. Available from: https://datainnovationproject.org/wp-content/uploads/2017/04/4_How_to_Conduct_a_Focus_Group-2-1.pdf. Accessed January 18, 2020.

[21] Bloor M, Frankland F, Thomas M, Robson K. Introducing Qualitative Methods: Focus Groups in Social Research. London: SAGE Publications Ltd; 2001.

[22] Mulhall A (2003). In the field: notes on observation in qualitative research. J Adv Nurs.

[23] Braun V, Clarke V, Terry G (2014). Thematic analysis. J Qual Res Clin Health Psychol.

[24] Graneheim UH, Lundman B (2004). Qualitative content analysis in nursing research: concepts, procedures and measures to achieve trustworthiness. Nurse Educ Today.

[25] Koly KN, Biswas T, Islam A (2015). Increasing Prevalence of Hypertension in Bangladesh: A review. Cardiovasc J.

[26] Kofi J. Prevention and management of Hypertension: a study on knowledge and attitudes of women of childbearing age [thesis]. Finland: Central Ostrobothnia University of Applied Sciences; 2011.

[27] Azhar S, Hassali MA, Ibrahim MI, Ahmad M, Masood I, Shafie AA (2009). The role of pharmacists in developing countries: the current scenario in Pakistan. Hum Resour Health.

[28] Miller R, Goodman C (2016). Performance of retail pharmacies in low- and middle-income Asian settings: a systematic review. Health Policy Plan.

[29] Demaio AR, Otgontuya D, de Courten M, Bygbjerg IC, Enkhtuya P, Meyrowitsch DW (2013). Hypertension and hypertension-related disease in Mongolia; findings of a national knowledge, attitudes and practices study. BMC Public Health.

[30] Tabassum N, Ahmad F (2011). Role of natural herbs in the treatment of hypertension. Pharmacogn Rev.

[31] Reinhart KM, Coleman CI, Teevan C, Vachhani P, White CM (2008). Effects of garlic on blood pressure in patients with and without systolic hypertension: a meta-analysis. Ann Pharmacother.

[32] Shaikh RB, Mathew E, Sreedharan J, Muttappallymyalil J, Sharbatti SA, Basha SA (2011). Knowledge regarding risk factors of hypertension among entry year students of a medical university. J Family Community Med.

[33] Ali S, Sathiakumar N, Delzell E (2006). Prevalence and socio-demographic factors associated with tobacco smoking among adult males in rural Sindh, Pakistan. Southeast Asian J Trop Med Public Health.

[34] Mannan MA (2013). Access to public health facilities in Bangladesh: a study on facility utilisation and burden of treatment. Bangladesh Dev Stud.

[35] Legido-Quigley H, Camacho Lopez PA, Balabanova D, Perel P, Lopez-Jaramillo P, Nieuwlaat R (2015). Patients’ knowledge, attitudes, behaviour and health care experiences on the prevention, detection, management and control of hypertension in Colombia: a qualitative study. PLoS One.

[36] Connell P, McKevitt C, Wolfe C (2005). Strategies to manage hypertension: a qualitative study with black Caribbean patients. Br J Gen Pract.

[37] Rousham EK, Khandakar IU (2016). Reducing health inequalities among girls and adolescent women living in poverty: the success of Bangladesh. Ann Hum Biol.

[38] El Arifeen S, Christou A, Reichenbach L, Osman FA, Azad K, Islam KS (2013). Community-based approaches and partnerships: innovations in health-service delivery in Bangladesh. Lancet.

[39] Rahman M, Nakamura K, Seino K, Kizuki M (2013). Does gender inequity increase the risk of intimate partner violence among women? evidence from a national Bangladeshi sample. PLoS One.

